# An Ensemble Framework for Projecting the Impact of Lymphatic Filariasis Interventions Across Sub-Saharan Africa at a Fine Spatial Scale

**DOI:** 10.1093/cid/ciae071

**Published:** 2024-04-25

**Authors:** Panayiota Touloupou, Claudio Fronterre, Jorge Cano, Joaquin M Prada, Morgan Smith, Periklis Kontoroupis, Paul Brown, Rocio Caja Rivera, Sake J de Vlas, Sharmini Gunawardena, Michael A Irvine, Sammy M Njenga, Lisa Reimer, Fikre Seife, Swarnali Sharma, Edwin Michael, Wilma A Stolk, Rachel Pulan, Simon E F Spencer, T Déirdre Hollingsworth

**Affiliations:** School of Mathematics, University of Birmingham, Birmingham, United Kingdom; CHICAS, Lancaster University, Lancaster, United Kingdom; Expanded Special Project for Elimination of Neglected Tropical Diseases (ESPEN), WHO Regional Office for Africa, Brazzaville, Democratic Republic of the Congo; School of Veterinary Medicine, University of Surrey, Guildford, United Kingdom; Department of Biological Sciences, University of Notre Dame, Notre Dame, Indiana, USA; Erasmus MC, University Medical Center Rotterdam, Rotterdam, The Netherlands; Zeeman Institute for Systems Biology and Infectious Disease Epidemiology Research, University of Warwick, Coventry, United Kingdom; Center for Global Health Infectious Disease Research, University of South Florida, Tampa, USA; Erasmus MC, University Medical Center Rotterdam, Rotterdam, The Netherlands; Department of Parasitology, University of Colombo, Colombo, Sri Lanka; Data and Analytic Services, British Columbia Centre for Disease Control, Vancouver, Canada; Eastern and Southern Africa Centre of International Parasite Control, Kenya Medical Research Institute (KEMRI), Nairobi, Kenya; Department of Vector Biology, Liverpool School of Tropical Medicine, Liverpool, United Kingdom; Disease Prevention and Control Directorate, Federal Ministry of Health, Addis Ababa, Ethiopia; Department of Mathematics, Vijaygarh Jyotish Ray College, Kolkata, India; Center for Global Health Infectious Disease Research, University of South Florida, Tampa, USA; Erasmus MC, University Medical Center Rotterdam, Rotterdam, The Netherlands; Faculty of Infectious and Tropical Diseases, London School of Hygiene & Tropical Medicine, London, United Kingdom; Zeeman Institute for Systems Biology and Infectious Disease Epidemiology Research, University of Warwick, Coventry, United Kingdom; Big Data Institute, Li Ka Shing Centre for Health Information and Discovery, University of Oxford, Oxford, United Kingdom

**Keywords:** fine-scale spatial projections, lymphatic filariasis, linking maps with models, ensemble models, intervention impact

## Abstract

**Background:**

Lymphatic filariasis (LF) is a neglected tropical disease targeted for elimination as a public health problem by 2030. Although mass treatments have led to huge reductions in LF prevalence, some countries or regions may find it difficult to achieve elimination by 2030 owing to various factors, including local differences in transmission. Subnational projections of intervention impact are a useful tool in understanding these dynamics, but correctly characterizing their uncertainty is challenging.

**Methods:**

We developed a computationally feasible framework for providing subnational projections for LF across 44 sub-Saharan African countries using ensemble models, guided by historical control data, to allow assessment of the role of subnational heterogeneities in global goal achievement. Projected scenarios include ongoing annual treatment from 2018 to 2030, enhanced coverage, and biannual treatment.

**Results:**

Our projections suggest that progress is likely to continue well. However, highly endemic locations currently deploying strategies with the lower World Health Organization recommended coverage (65%) and frequency (annual) are expected to have slow decreases in prevalence. Increasing intervention frequency or coverage can accelerate progress by up to 5 or 6 years, respectively.

**Conclusions:**

While projections based on baseline data have limitations, our methodological advancements provide assessments of potential bottlenecks for the global goals for LF arising from subnational heterogeneities. In particular, areas with high baseline prevalence may face challenges in achieving the 2030 goals, extending the “tail” of interventions. Enhancing intervention frequency and/or coverage will accelerate progress. Our approach facilitates preimplementation assessments of the impact of local interventions and is applicable to other regions and neglected tropical diseases.

## Teaser

The integration of a geospatial statistical model with an ensemble of 3 transmission models of lymphatic filariasis enables the projection at a fine spatial scale of the expected progress toward disease elimination under various intervention scenarios across sub-Saharan African countries.

Lymphatic filariasis (LF) is a neglected tropical disease (NTD) caused by infection with mosquito-borne parasites. The parasites dwell in the lymphatic system, leading to impaired lymph flow and eventually lymphoedema, elephantiasis, or hydrocele. For LF, the World Health Organization (WHO) called for its elimination as a public health problem (EPHP) in 1997 and launched the Global Programme to Eliminate Lymphatic Filariasis in 2000 [1]. The aims of the global program are to interrupt transmission by annually treating entire at-risk populations with antifilarial drugs (termed *mass drug administration* or *MDA*) and to alleviate the suffering of people already affected by filarial disease by providing disease management and hygienic measures [1].

More recently, WHO launched the road map for NTDs in 2021–2030 [2], which sets a target for LF of EPHP in 58 (81%) of 72 endemic countries worldwide by 2030, while the remaining endemic countries (14 countries [19%]) should have completed their MDA programs and be in the posttreatment surveillance phase to validate elimination. MDA can be stopped if, after ≥5 rounds of MDA with good coverage (65% of the entire population receiving MDA treatment), the infection prevalence has dropped below the transmission assessment survey (TAS) thresholds set by WHO [3]. In sub-Saharan Africa, where *Wuchereria bancrofti* is endemic and *Anopheles* is the dominant vector of transmission, these thresholds effectively mean that prevalence of the disease must be below 1% microfilaria (mf) or 2% antigenemia in populations >5 years of age. Elimination can be validated by demonstrating that prevalence is sustained below this threshold in 2 repeated surveys, to be performed approximately 2–3 years after the previous survey [3].

Despite the enormous progress made since the inception of the Global Programme to Eliminate Lymphatic Filariasis [4], there is a large amount of heterogeneity across sub-Saharan African countries in the progress to elimination, owing to several factors like variation in transmission conditions and in the implementation of MDA. For example, prevalence levels before the introduction of control programs vary significantly across regions, with some reporting precontrol prevalence as low as 0,% while others experience rates as high as 40% [5 ,6]. Implemented interventions also differ widely between and within countries. For instance, countries like Nigeria or Togo have had MDA programs running since the year 2000, while others like South Sudan or São Tomé and Príncipe started MDA only recently [7].

Different treatment regimens are also used across the continent, depending on coendemicity of other filarial infections. Diethylcarbamazine plus albendazole is globally the preferred treatment regimen, which can also be combined with ivermectin to further boost efficacy. However, diethylcarbamazine cannot safely be used in onchocerciasis-endemic regions, so ivermectin plus albendazole is the mainstay of treatment in most of the African region. Neither diethylcarbamazine nor ivermectin can be used in loiasis-coendemic areas, which instead receive twice-yearly albendazole. Bed nets, which are primarily used for malaria control, can also contribute to the control of LF by reducing exposure to mosquito bites during peak transmission periods when infected mosquitoes are actively biting humans. The combined use of bed nets with MDA has been shown to reduce transmission by *Anopheles* [8, 9], and it is possible that vector control alone may be sufficient to achieve elimination, as suggested in the Gambia, where bed net programs alone were enough to interrupt *W. bancrofti* transmission [10]. Variation in bed net coverage, as well as the combination with MDA or not, contributes to further heterogeneity between locations [11].

Finally, the achieved coverage of MDA programs, which should be at least 65% per round, also varies across locations. As a result, the required treatment duration and expected end year of interventions vary strongly. In some areas, 5 or 6 annual MDA rounds have been sufficient to interrupt transmission, while in other areas transmission persisted even after >10 years of MDA efforts [4]. Considering this heterogeneity in prevalence and intervention histories across African countries and regions, there is increasing interest in developing strategies that can integrate geographic information to help in evaluating the potential effectiveness of elimination programs for LF across regions and to explore potential alternatives that can be used to accelerate progress in specific areas where elimination proves challenging.

Mathematical models of NTDs have been used to inform public health policy for many years [12]. One approach commonly used is scenario-based modeling, which provides broad insights by defining generic settings. An example of this is the recent work modelling the delays in LF programs owing to the coronavirus disease 2019 (COVID-19) pandemic [13]. However, defining general scenarios means that the nuance of specific countries or subnational regions is not captured, such as their history of MDA programs and precontrol endemicity levels. Studies have highlighted the significance of addressing spatial heterogeneities in disease transmission dynamics [14, 15] and the need to address such heterogeneous dynamics for minimizing aggregation error when making predictions at a coarse scale [16]. For example, Michael et al [16] developed a spatially hierarchical data-driven computational platform to tackle the problem of scaling up from local settings and enable predictions at regional levels by the discovery and use of locality-specific transmission models. Their findings contrast with previous national-level intervention modeling approaches, highlighting the need to account for heterogeneous transmission dynamics across a spatial domain.

In the current study, we use a bayesian approach that combines fine-scale geostatistical maps of LF prevalence before MDA initiation with an ensemble of 3 disease transmission models, to facilitate the investigation of policy questions related to LF elimination in sub-Saharan Africa. While each model offers valuable insights independently, we use an approach that combines the 3 individual models to generate fine-scale ensemble projections. More specifically, we use this approach to forecast progress toward the 2030 goals using current interventions [17, 18], in order to provide prevalence estimates at fine spatial scales from 2021 to 2030 and identify areas where alternative interventions may be needed to accelerate progress toward elimination by 2030. Furthermore, we estimate the likely impact of such alternative interventions, including those that rely on increased MDA frequency or coverage. By assessing the impact of different strategies on LF elimination timelines across distinct areas, the proposed methods can serve as a valuable tool for policy makers to optimize interventions and ensure the effective control of LF tailored to local context.

## METHODS

### Pre-MDA Geostatistical Map

The starting point for the analysis was a pre-MDA geostatistical map of infection prevalence, which was generated through the analysis of 2 markers: mf and antigenemia. As the first marker, mf are found in the blood of infected individuals and can be used as an indicator of transmission, as mosquitoes can ingest mf during blood meals from infected hosts. The mf diagnostic is also found more frequently in historic data. However, there are disadvantages associated with the use of mf counts, as these require specialized parasitological skills and night sampling (to capture parasite activity in the infected host). By contrast, antigenemia is easier to assess by using an antigenic immunochromatographic card test (ICT), which measures antigenic activity against adult parasites. In recent years, ICT has been mostly replaced by the filarial test strip, which is now the WHO-recommended diagnostic method for LF mapping, monitoring, and evaluation [19]. Since the transmission models used in this study have been more extensively validated against mf data than against ICT data, and our primary focus is on historic data, which are more frequent in the early years of the program, we generated a geostatistical estimate of mf prevalence.

We started by generating a 5 × 5-km scale pixel map of mf prevalence across sub-Saharan African countries before interventions, using a model-based geostatistical approach (detailed in the Supplementary Materials). Because the LF transmission models were calibrated using mf prevalence, we converted ICT prevalence to mf prevalence, as reported elsewhere [20]. Given that the diagnostic tests are used only in individuals aged ≥5 years, the resulting map represents LF prevalence within this specific subpopulation. Furthermore, the map reflects the LF prevalence status at the baseline, which refers to the time point before the initiation of MDA, termed here as *precontrol prevalence* (Supplementary Figure SG10 in the Supplementary Materials).

### Combining Geostatistical Map With Transmission Models and Intervention Histories

The precontrol geostatistical map was then linked to transmission models of LF through fitting of the models to the pixel level data, based on recently developed methods [21]. We used 3 published mathematical models of LF transmission and control: EPIFIL [22–25], a deterministic population-based model, and LYMFASIM [26–28] and TRANSFIL [29, 30], 2 stochastic individual-based model. The 3 models were used to estimate the impact of historical, current and future interventions, including vector control (bed nets) and MDA with different drug combinations, depending on the area.

The first step of the method is to generate a large number of simulations from the transmission models, which encompass the entire range of prevalences observed in the geostatistical model. This can be achieved by defining the parameters that spatially vary across Africa, such as vector density and aggregation, and drawing them from a prior distribution informed from data, pilot simulations, and previous analyses (see the Supplementary Materials for a complete overview of all parameter values and prior distributions for each model). An ensemble modeling approach is then used, in which simulations from all individual models are combined together to formulate a collective ensemble of simulations. These ensemble simulations are then weighted according to how closely they match the prevalence distribution and population size of each pixel. Population data for each pixel are extracted from the WorldPop website [31]. Finally, once the weights are calculated, the transmission models are run forward in time, considering the history of control in each pixel, to obtain future estimated distribution of projections across space.

Data on historical MDA campaigns in each region were obtained from the Expanded Special Project for Elimination of Neglected Tropical Diseases (ESPEN) website [32]. To reduce the number of alternative treatment histories modeled, we made several conservative assumptions. First, for MDA coverage, pixels were classified within regions according to year-by-year MDA coverage estimates, taking the true coverage to be 65% if the reported coverage exceeded this value, 15% if the reported value was between 15%–65%, and zero otherwise. Second, to incorporate the contribution of vector control, we used the coverage data of insecticide-treated bed nets, which can be extracted from the Malaria Atlas Programme [11]. We classified this coverage into 4 different bins: 0%–24%, 25%–49%, 50%–74%, and 75%–100%. Note that in the current study we do not account for insecticide resistance in *Anopheles* mosquitoes when modeling bed net efficiency. However, we acknowledge the importance of this factor and plan to consider it in future work.

**Figure 1.**
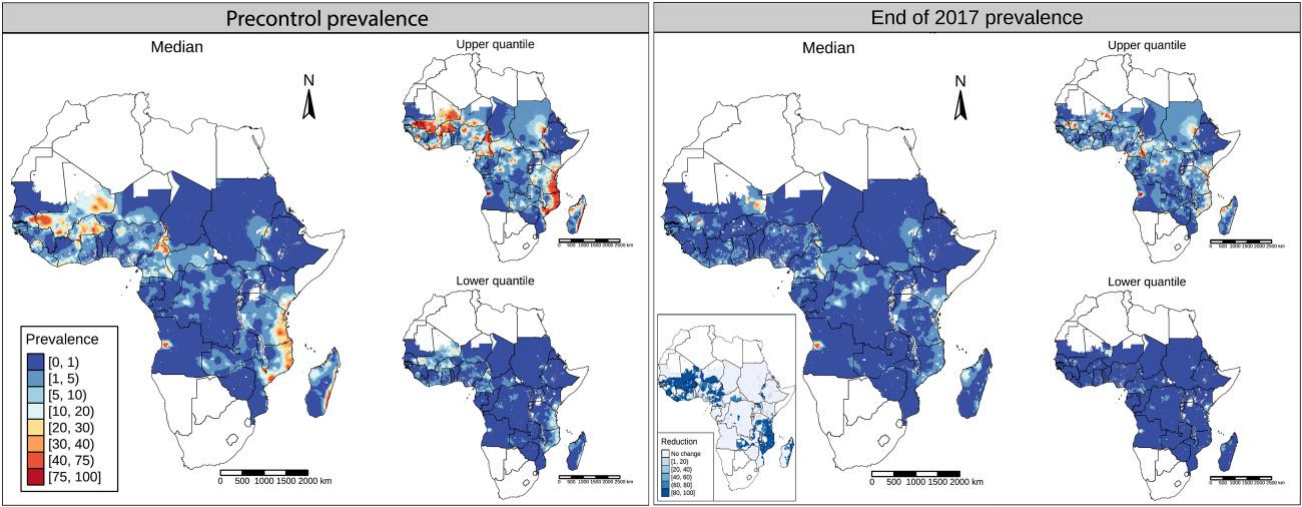
Estimated median baseline microfilaria (mf) prevalence (*left panel*) and estimated median prevalence by the end of 2017 (*right panel*) at the pixel level (5 × 5 km^2^ squares). Upper (97.5%) and lower (2.5%) quantiles are shown on the right-hand side in each panel. Inset in right panel shows the change in the median prevalence, per pixel, between precontrol and 2017 prevalence.

In addition to MDA and bed net coverage, we also accounted for the year that MDA started and duration of the program (see Supplementary Figure S9 in the Supplementary Materials). At the time of this analysis, complete historical information in interventions was available up to 2017. This information was available at the implementation unit (IU) level, which represents either the first or the second level administrative unit, depending on each country. We assumed that programs that started before 2017 would continue, and we included MDA programs starting in 2018–2019 in the IUs where such data was available. Otherwise, we assumed that programs in all remaining IUs started in 2020. Note that areas with estimated prevalence levels <1% mf (the target threshold for EPHP) by the end of 2017 (as indicated in the right panel of Figure 1), were considered nonendemic and were excluded from subsequent analyses. Consequently, forward simulations were carried out only in areas classified as endemic in 2018.

For all programs that (re)start or continue after 2017, three possible intervention strategies at the IU level were considered: (1) long term, with continuation of current interventions, generally consisting of annual MDA with 65% coverage; (2) increased coverage, in which the IU increases MDA coverage to 80%; and (3) increased frequency, in which annual MDA is increased to biannual (2 rounds per year). As mentioned above, the drug combinations that countries use may vary, including albendazole alone, albendazole paired with ivermectin or diethylcarbamazine, or a combination of all 3 drugs (see Supplementary Figure S10 in the Supplementary Materials). Considering all the various backgrounds of historical treatments (MDA and/or bed nets) and drugs used, the total number of scenarios simulated was 2611.

For each scenario, 100 000 simulations were generated from each of the 3 transmission models, using parameter values drawn from their prior distributions, to ensure that there was sufficient diversity in the runs to accurately capture the dynamics across all spatial locations under consideration. Adopting an ensemble approach, the simulations from the 3 models were combined into a single set, resulting in a total of 300 000 simulations. These ensemble simulations were then weighted according to how closely they matched the characteristics of each pixel, such as population size and baseline prevalence, as described elsewhere [21].

### Ensemble Predictions

Once the simulations were matched to the pixels at baseline, we simulated the transmission models forward in time, under the 3 possible future intervention strategies described above, to determine the projected prevalence outcome locally (ie, by pixel). Since the campaigns were deployed at an IU level, the results from the ensemble model were aggregated for each IU by taking the average of the 90% quantile of the prevalence distributions of the pixels in it, weighted by population size. Therefore, we consider that an IU achieves the minimum condition to start validation of EPHP once this average falls below the TAS threshold (1% mf prevalence). Conversely, any IUs in which this average was above the TAS threshold were considered not to have achieved the EPHP condition.

## RESULTS

In this analysis, we start by describing the estimation of a baseline map that captures LF prevalence across sub-Saharan African countries at the fine scale before the introduction of MDA campaigns. The transmission models were then used to produce simulations that match this precontrol map, before being run forward in time, accounting for all the historical treatment that has taken place until 2017.

Originally, one key objective of this project was to estimate the likelihood that different regions would achieve the elimination targets set out in the 2012–2020 WHO NTD road map [33]. However, WHO has since released revised targets for LF elimination by 2030 in its new road map for NTDs 2021–2030 [2]. Therefore, we further extended our predictions under a range of potential intervention strategies between 2018 to 2030, to identify regions where additional measures may be useful in accelerating progress toward LF elimination (see Methods for details).

Our findings show that before the implementation of control programs (estimated precontrol prevalence map; Figure 1, *left*), mf prevalence across sub-Saharan Africa was very heterogeneous. For example, while most pixels have mf incidences <1% (ie, are nonendemic), some pixels have estimated prevalences above 75%. By the time this analysis was performed, complete treatment histories were available up to 2017. When these are used to project mf prevalence from the baseline up to 2017, the map changes significantly. For example, only very few high prevalence pixels remain in the prevalence map at the end of 2017 (Figure 1, *right*). For some countries, such as Mali, Burkina Faso, or Mozambique, we find significant changes in estimated median mf prevalence between the baseline and 2017 maps, with some areas in these countries achieving reductions above 80% (Figure 1, inset right). However, despite such important reductions and although most pixels (66%) have an estimated median prevalence below 1% in 2017, our results reveal some areas of high endemicity, suggesting that the historical rounds are not sufficient to ensure that LF will have achieved EPHP status across the whole region. Notably, projections for some of these areas, such as Kenya, are characterized by high uncertainty, which is likely due to uncertainty linked to the baseline estimates of LF prevalence.

**Figure 2.**
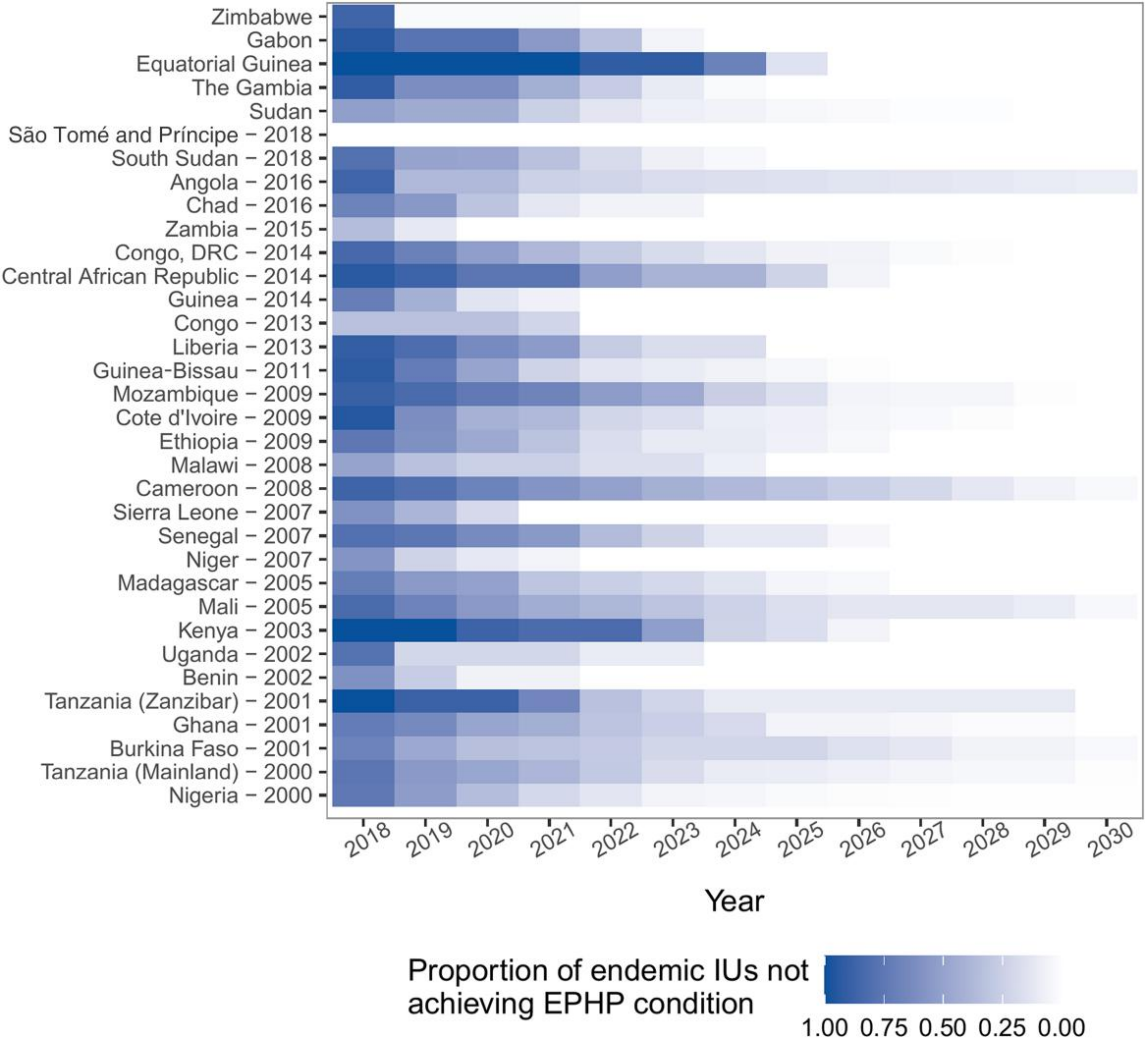
Proportion of implementation units (IUs) not achieving elimination as a public health problem (EPHP) condition, estimated from the number of IUs with a <90% probability of being under the EPHP threshold. IUs are grouped by country (y-axis), with the start year of the program in each country also highlighted. Countries without a specified start year in the top panel had not started mass drug administration (MDA) by the end of 2017 (time frame for which we had data available). For these countries, we assume that MDA began in 2020.

For regions that were endemic by the end of 2017, we then carried out forward simulations under a range of scenarios, to determine when different IUs would achieve EPHP status. First, we modeled the likely impact of continuing to use the same interventions from 2018 onward. For this, we used the drug(s) of choice in each country and assumed a 65% coverage achieved with annual rounds of treatment, except for some IUs in Angola, Central African Republic, Cameroon, Democratic Republic of the Congo, Gabon, Equatorial Guinea, Nigeria, South Sudan, and Chad, where biannual rounds are expected to be carried out. Our simulations suggest that under this scenario most of sub-Saharan Africa will likely reduce mf prevalence below the EPHP threshold of 1% mf by 2030 with 90% probability (Figure 2). Our simulations suggest that by 2030 only 6 countries (Angola, Nigeria, Tanzania [Mainland], Burkina Faso, Mali, and Cameroon) may still have some IUs that fail to meet the EPHP condition. Furthermore, we find that many countries are likely to achieve EPHP before 2030, with 16 of 34 countries estimated to have reached prevalence below 1% mf in all IUs by 2026 (Figure 2).

**Figure 3.**
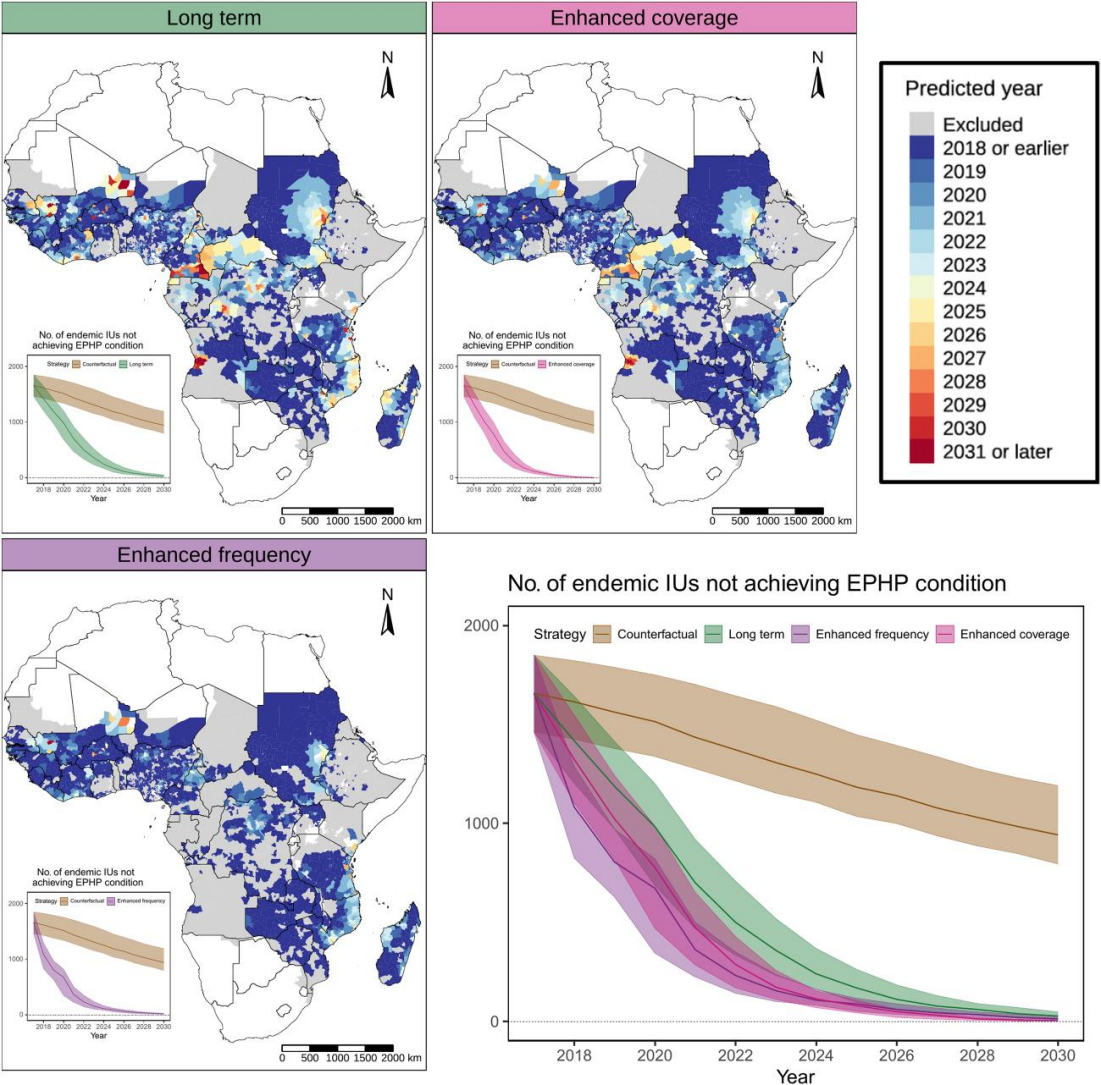
Year of achieving the elimination as a public health problem (EPHP) condition across the implementation units (IUs) assessed. Gray areas were excluded, either because they are nonendemic or, in the enhanced frequency map, because the long-term intervention is already biannual (and it is thus not appropriate to increase frequency). Inset panels show the number of endemic IUs not achieving the EPHP condition over time. Counterfactual is defined as the scenario in which mass drug administration is halted in 2018.

We also simulated how alternative interventions are likely to affect the timeline to LF elimination across the different areas. For this, we compared the long-term intervention described above (mainly relying on annual MDA campaigns with 65% coverage) with improved interventions relying on increased coverage (80%) or frequency (biannual). The timeline for achieving the condition for EPHP was then estimated as the first year in which the probability of being below the TAS threshold is >90% (Figure 3).

Our findings show that such improved interventions can accelerate progress toward LF elimination. For example, while 15 IUs were estimated not to achieve the EPHP condition with the current strategy applied long term by 2030, only one of them fails to meet this target when higher MDA coverage or frequency are used (Figure 3, *top right and bottom left*). As expected, our results show that changing the current MDA strategy is mostly needed in IUs estimated to have high prevalence at the baseline (Figure 1). Many of these IUs are also characterized by large uncertainty in the geospatial map (eg, those in Mali and Kenya) and sometimes also characterized by limited treatment histories (eg, Sudan), further increasing uncertainty.

Moreover, our findings indicate that increasing the intervention frequency to biannual or enhancing the coverage to 80% can accelerate progress by up to 5 or 6 years (Figure 3*, bottom right* ). In a comparison of the 2 modeled acceleration scenarios, the biannual MDA at 65% coverage is identified as the most efficient, attaining elimination more quickly than the annual MDA with 80% coverage.

## DISCUSSION

Our findings demonstrate the utility of integrating historical geostatistical data on LF prevalence with disease transmission models to better understand the likely impact of different interventions on progress toward LF elimination at a fine spatial scale. A key finding from our analysis is that current interventions are highly efficacious in reducing LF endemicity across most geographies, as highlighted by estimated reductions in the median prevalence in some areas above 80% (Figure 1*, right inset* ). These data agree with epidemiological data showing that existing interventions have driven major progress toward control and elimination of LF in many countries, with a 36% reduction in the population requiring MDA since 2000 [34].

Looking forward, even with conservative assumptions and relying on current interventions, progress in the continent is likely to lead to achieving the infection threshold for EPHP before 2030 in 29 of the 34 endemic countries in Africa (Figure 2). The rationale for the TAS threshold is that it would effectively eliminate the incidence of disease, including lymphodema and hydrocele, and it is paired with criteria on effective programs to treat and alleviate the burden of disease. In our study we considered only infection, owing to the challenges of quantifying the link between infection and disease, and we did not consider surgery or other treatment programs to alleviate disease.

It is also important to note that in the WHO road map for NTDs 2021–2030, the objective for LF is validation of EPHP, which requires countries to be below the TAS threshold at least 4 years after stopping MDA (independently of whether it is measured as a 1% mf threshold, as used here, or a 2% antigenemia threshold). Here, we assessed only the probability of different IUs reaching the TAS threshold, rather than remaining below it once MDA is interrupted. IUs that we have considered meet the EPHP condition would still need to maintain those prevalence levels for 4 years in the absence of MDA, so with our definition, only areas achieving the EPHP condition before 2026 could be validated by 2030. In our analysis, 16 of the 34 endemic countries meet our EPHP condition by 2026 and would thus be very likely to pass pre-TAS and achieve validation (Figure 2). It is also important to note that we use a very strict definition of the EPHP condition, with the need for an IU to have a ≥90% probability of mf prevalence being <1%. This means that IUs that we consider have not reached the EPHP condition by a certain date might still be able to pass pre-TAS.

While we find that most countries and regions are on track to meet the 2030 targets using current strategies, we also identified 15 IUs where simply prolonging these interventions will be insufficient to achieve EPHP by 2030. By modeling the impact of enhanced interventions with increased MDA frequency (biannual vs annual) or coverage (80% vs 65%), we found that progress toward LF elimination can be accelerated. Of note, our simulations show that the EPHP condition would likely be met in all but 1 IU (in Mali) under these alternative scenarios (Figure 3). Our analysis indicates that the acceleration scenario of increasing MDA frequency to biannual showed more rapid progress than enhancing the coverage to 80%. However, it is essential to underline that these strategies come with distinct operational challenges and cost implications. These critical factors should be considered and integrated within the local context to ascertain the most feasible and effective implementation strategy. Tailoring the approach to the unique circumstances and constraints of each location is essential to optimizing the impact and efficacy of the intervention strategies.

Furthermore, our results, which agree with those of previous studies showing the potential benefits of increasing MDA coverage and/or frequency [16, 18], demonstrate that the 2030 target of eliminating LF across sub-Saharan Africa is feasible, although some additional efforts may be needed in regions of historically high endemicity. This aligns with studies indicating that the success of elimination programs can be influenced by a wide range of factors, such as initial levels of endemicity, the specific MDA regimen used, treatment frequency, duration, and population coverage, among others [35].

While the delays due to COVID-19 to some national programs have not been considered here, recent work suggests that the consequences might be limited, particularly if activities can be resumed quickly [13, 36, 37]. Undoubtedly, programs will be facing many challenges in the coming years, including reduced domestic revenues, cuts to funding from donors, and changes in transmission linked to climate change. Nonetheless, our findings not only highlight the great progress toward LF elimination made to date but also provide optimistic expectations for the future, demonstrating that while elimination of LF is an ambitious goal, it is certainly possible.

The main limitation of our approach is that we only model different scenarios from historical baseline mf prevalence before the start of interventions. Therefore, the accuracy and relevance of our approach can be improved by incorporating more contemporary data on LF prevalence, although obtaining such data across such a wide range of countries and regions has associated challenges. Furthermore, data scarcity for some IUs results in high uncertainty in their prevalence estimates, which influences our projections and the determination of the likelihood that such areas will fulfill the EPHP condition.

A key limitation of our modeling approach is the exclusion of insecticide resistance in *Anopheles* mosquitoes, a factor that has been increasingly recognized as a critical element in the efficacy of vector control strategies. For example, Hemingway et al [38] showed that bed net coverage does not necessarily equate to effective transmission reduction in areas with prevalent resistance. In future work, we aim to incorporate insecticide resistance mapping with LF pixel prevalence data, enabling us to tailor recommendations for LF control more effectively by accounting for local variations in insecticide resistance and its impact on transmission dynamics.

Despite these limitations, our analysis demonstrates that the approach used here, which combines geospatially resolved prevalence data with an ensemble of transmission models, can be a powerful tool in evaluating the effectiveness of proposed intervention strategies at a fine spatial scale. By capturing the complexities of disease transmission and incorporating spatial information, our framework can account for differences in local context and provide valuable insights into the potential effects of interventions, thereby more effectively supporting evidence-based decision making before the implementation of control and elimination measures.

## Supplementary Data

Supplementary materials are available at *Clinical Infectious Diseases* online. Consisting of data provided by the authors to benefit the reader, the posted materials are not copyedited and are the sole responsibility of the authors, so questions or comments should be addressed to the corresponding author.

## Supplementary Material

ciae071_Supplementary_Data

## Notes

***Author contributions.*** Conceptualization: P. T., C. F., J. M. P., S. G., S. M. N., F. S., R. P., S. E. F. S., and T. D. H . Data creation: P. T., C. F., J. M. P., and S. G. Formal analysis: P. T., C. F., J. M. P., S. E. F. S., and T. D. H. Funding acquisition: T. D. H. Investigation, methodology, and software: P. T., J. M. P., P. B., M. I., S. E. F. S., and T. D. H (TRANSFIL); C. F., and R. P. (geospatial model); M. S., R. C. R., S. S., and E. M. (EPIFIL); P. K., S. J. d. V., and W. A. S. (LYMFASIM). Project administration: P. T., J. M. P., S. E. F. S., and T. D. H. Supervision: S. J. d. V., L. R., F. S., E. M., R. P., S. E. F. S., and T. D. H. Validation: P. T. and J. M. P. Visualization: P. T., C. F., J. M. P., and T. D. H. Writing original draft: P. T., and J. M. P. Writing, review, and editing: all authors.

***Acknowledgments.*** The authors gratefully acknowledge the contributions of Claudio Nunes Alves in reviewing this manuscript. They also thank Matt Keeling for helpful discussions.

***Financial support.*** The authors are grateful for funding by the Bill and Melinda Gates Foundation through the NTD Modelling Consortium (grants OPP1186851, OPP1156227). This supplement is sponsored by funding of Professor T. Déirdre Hollingsworth's research by Li Ka Shing Foundation at the Big Data Institute, Li Ka Shing Centre for Health Information and Discovery, University of Oxford and funding of the NTD Modelling Consortium by the Bill and Melinda Gates Foundation (grant INV-030046)

***Supplement sponsorship.*** This article appears as part of the supplement “New Tools and Nuanced Interventions to Accelerate Achievement of 2030 Roadmap for Neglected Tropical Diseases,” sponsored by funding of Professor T. Déirdre Hollingsworth's research by the Li Ka Shing Foundation at the Big Data Institute, Li Ka Shing Centre for Health Information and Discovery, University of Oxford; and funding of the NTD Modelling Consortium by the Bill & Melinda Gates Foundation (INV-030046).
