# Supplementary material for: An Ensemble Framework for Projecting the Impact of Lymphatic Filariasis Interventions Across Sub-Saharan Africa at a Fine Spatial Scale
Source: Clin Infect Dis. 2024 Apr 25;78(Suppl 2):S108–16. doi: 10.1093/cid/ciae071 (PMC11045016; doi:10.1093/cid/ciae071)
Supplement: ciae071_Supplementary_Data [file ciae071_supplementary_data.zip › Supplementary Geostatistical Model.pdf]

Supplementary Material: Geostatistical Model

P. Touloupou, C. Fronterre et al.

This document summarise descriptive information about the data used in the paper and the geostatistical modelling approach developed to convert ICT prevalence to MF prevalence.

Keywords: lf, geostatistics, prevalence

1 Tables

Table SG 1: Summary of parasitological data used in mapping.

			# of persons examined		Cases detected		Prevalence (%)		
**Country**	**Year**	**# survey sites**	**Total**	**Median**	**Total**	**Median**	**Minimum**	**Median**	**Maximum**
Angola	1966	2	591	296	0	0	0.0	0.0	0.0
Benin	1954	1	62	62	0	0	0.0	0.0	0.0
Benin	1967	2	301	150	1	0	0.0	0.2	0.4
Benin	1968	16	2360	138	298	20	0.0	10.7	39.4
Benin	1983	3	365	134	34	13	4.1	7.1	14.2
Benin	1994	4	410	90	2	0	0.0	0.3	1.4
Burkina Faso	1965	29	8783	152	519	8	0.0	7.7	35.3
Burkina Faso	1966	11	1465	130	413	35	18.5	27.7	46.4
Burkina Faso	1967	4	1068	144	373	61	2.9	24.5	73.5
Burkina Faso	1968	149	59246	275	7594	15	0.0	7.0	42.7
Burkina Faso	1969	10	3703	200	1147	76	6.8	38.0	48.2
Burkina Faso	2002	6	1043	159	34	2	0.5	1.5	7.2
Cameroon	1957	11	1725	120	199	4	0.0	2.7	20.1
Cameroon	1984	1	1000	1000	4	4	0.4	0.4	0.4
Cameroon	1998	2	495	248	71	36	11.9	14.5	17.0
Cameroon	2002	9	995	96	9	0	0.0	0.0	3.2
Cameroon	2006	9	714	74	0	0	0.0	0.0	0.0
Cameroon	2007	11	5500	500	66	6	1.0	1.2	2.0
Chad	1970	5	1681	363	38	0	0.0	0.0	9.1
Comoros	1969	9	1607	147	663	55	26.5	40.5	56.7
Comoros	1982	6	401	75	75	8	9.9	16.0	29.0
Congo (Brazzaville)	2012	1	774	774	41	41	5.3	5.3	5.3
Congo (Kinshasa)	1946	48	3244	50	102	0	0.0	0.0	36.1
Congo (Kinshasa)	1968	8	648	96	4	0	0.0	0.0	2.0
Congo (Kinshasa)	1970	32	2474	80	18	0	0.0	0.0	32.1
Cote d'Ivoire	1965	22	3794	120	189	2	0.0	3.0	41.7
Ethiopia	1971	19	1368	51	45	0	0.0	0.0	50.0
Ethiopia	1973	2	82	41	33	16	29.3	40.2	51.2
Ethiopia	2005	4	205	51	0	0	0.0	0.0	0.0
Ghana	1994	7	2968	418	329	4	0.0	1.5	25.4
Ghana	1996	1	296	296	78	78	26.4	26.4	26.4
Ghana	1997	15	1562	106	266	12	1.2	14.8	39.6
Ghana	2001	22	2215	100	509	22	10.1	21.0	52.0
Ghana	2002	8	941	66	43	2	0.0	2.8	14.0
Guinea	1954	2	732	366	14	7	0.0	2.0	4.0
Kenya	1962	13	5290	434	878	57	2.6	16.4	32.2
Kenya	1972	35	4265	63	1019	11	0.0	16.2	57.1
Kenya	1985	2	222	111	64	32	26.7	28.1	29.6
Kenya	1990	3	1716	570	281	72	12.1	12.6	24.9
Kenya	1994	12	6531	481	1047	86	8.1	15.4	27.4
Kenya	1998	3	2008	825	130	27	2.7	2.7	47.6
Kenya	1999	1	642	642	20	20	3.1	3.1	3.1

Table SG 1: Summary of parasitological data used in mapping. *(continued)*

Country	Year	# survey sites	Total	Median	Total	Median	Minimum	Median	Maximum
Kenya	2002	10	1620	176	315	32	2.8	20.1	22.9
Kenya	2004	1	304	304	67	67	22.0	22.0	22.0
Liberia	1947	1	193	193	25	25	13.0	13.0	13.0
Liberia	1948	17	9927	497	84	2	0.0	0.4	7.9
Liberia	1949	2	547	274	10	5	0.0	1.7	3.3
Liberia	1951	9	1206	68	22	2	0.0	3.6	5.8
Liberia	1971	11	615	52	72	5	0.0	11.5	36.6
Liberia	1972	75	9327	97	724	1	0.0	1.8	37.3
Liberia	1973	13	1340	99	204	14	5.4	17.6	26.4
Liberia	1976	8	1054	117	215	26	13.2	19.4	31.9
Liberia	1977	5	409	80	53	8	6.2	10.9	26.7
Liberia	1978	6	760	119	115	20	2.0	21.4	41.3
Madagascar	1950	1	377	377	152	152	40.3	40.3	40.3
Madagascar	1956	1	24	24	1	1	4.2	4.2	4.2
Madagascar	1970	1	3902	3902	359	359	9.2	9.2	9.2
Madagascar	1996	7	1340	188	303	51	7.8	27.1	32.6
Malawi	2000	2	537	268	116	58	20.3	21.5	22.6
Mali	1968	17	5320	226	596	18	0.8	4.8	27.0
Mali	1972	6	795	118	104	12	3.8	9.9	21.1
Mali	2000	1	460	460	7	7	1.5	1.5	1.5
Mali	2002	6	1139	202	244	38	13.8	18.8	40.0
Mozambique	1958	4	1420	260	108	28	4.2	8.8	13.7
Niger	1954	1	79	79	5	5	6.3	6.3	6.3
Niger	1967	5	831	169	4	0	0.0	0.0	1.5
Nigeria	1965	1	93	93	2	2	2.2	2.2	2.2
Nigeria	1978	2	847	424	235	118	25.4	27.4	29.3
Nigeria	1984	11	2196	136	121	10	2.2	7.3	11.8
Nigeria	1989	9	2485	233	276	29	5.2	11.0	27.3
Nigeria	1990	18	2689	110	46	2	0.0	1.8	5.3
Nigeria	1995	10	1503	94	20	2	0.0	1.3	3.3
Nigeria	2001	12	1736	108	128	8	0.0	5.5	21.6
Nigeria	2002	8	1007	100	183	19	7.4	15.0	38.6
Nigeria	2006	1	780	780	80	80	10.3	10.3	10.3
Nigeria	2008	6	897	155	139	16	1.3	12.6	30.3
Nigeria	2009	5	1007	221	48	10	0.0	5.8	8.5
Nigeria	2010	1	1803	1803	127	127	7.0	7.0	7.0
Senegal	1952	1	390	390	154	154	39.5	39.5	39.5
Senegal	1953	1	24	24	5	5	20.8	20.8	20.8
Senegal	1954	1	49	49	24	24	49.0	49.0	49.0
Senegal	1964	9	888	93	26	1	0.0	1.0	17.0
Senegal	1970	9	1841	173	434	49	11.0	28.5	33.8
Senegal	1975	6	801	140	238	30	13.2	36.1	64.4
Seychelles	1970	4	2782	401	188	40	3.5	6.0	17.1
Sierra Leone	1993	5	630	130	64	12	7.9	9.5	13.0
Tanzania (Mainland)	1952	14	3538	210	93	2	0.0	1.3	43.2
Tanzania (Mainland)	1966	1	161	161	17	17	10.6	10.6	10.6
Tanzania (Mainland)	1974	4	3532	594	987	126	18.5	26.3	32.0
Tanzania (Mainland)	1992	7	3353	467	897	139	17.7	28.5	34.7
Tanzania (Mainland)	1994	3	1704	604	198	84	6.1	12.8	14.4
Tanzania (Mainland)	1998	2	2794	1397	756	378	24.9	26.4	28.0
Tanzania (Mainland)	1999	1	622	622	183	183	29.4	29.4	29.4
Tanzania (Mainland)	2000	1	1018	1018	120	120	11.8	11.8	11.8
Tanzania (Mainland)	2004	1	919	919	225	225	24.5	24.5	24.5
Tanzania (Mainland)	2007	11	701	64	23	2	0.0	3.2	11.8
Tanzania (Zanzibar)	1965	1	110	110	8	8	7.3	7.3	7.3
Tanzania (Zanzibar)	1975	3	2760	905	392	128	11.8	15.1	16.2
Tanzania (Zanzibar)	1988	2	1324	662	394	197	10.5	30.0	49.5
Tanzania (Zanzibar)	1989	1	646	646	61	61	9.4	9.4	9.4
Tanzania (Zanzibar)	1990	3	2687	964	260	113	2.8	11.7	12.9
Tanzania (Zanzibar)	1992	1	274	274	103	103	37.6	37.6	37.6
The Gambia	1951	1	603	603	220	220	36.5	36.5	36.5
The Gambia	1952	3	795	255	217	82	19.2	25.9	38.4
The Gambia	1953	1	150	150	40	40	26.7	26.7	26.7

Table SG 1: Summary of parasitological data used in mapping. *(continued)*

Country	Year	# survey sites	Total	Median	Total	Median	Minimum	Median	Maximum
The Gambia	1976	15	1514	102	154	7	1.5	7.0	22.7
Togo	1968	6	566	87	36	4	0.0	3.4	13.5
Togo	1975	8	814	110	20	1	0.0	1.2	10.6
Uganda	1998	4	1430	372	238	62	0.0	16.1	25.5
Zimbabwe	1958	1	26	26	9	9	34.6	34.6	34.6
Zimbabwe	1970	2	109	54	4	2	2.6	4.4	6.2
Zimbabwe	1971	1	42	42	7	7	16.7	16.7	16.7

Table SG 2: Summary of serological data used in mapping.

			# of persons examined		Cases detected		Prevalence (%)		
Country	Year	# survey sites	Total	Median	Total	Median	Minimum	Median	Maximum
Angola	2015	116	10678	100	1350	0	0.0	0.0	98.0
Angola	2016	96	8096	99	66	0	0.0	0.0	19.2
Benin	2000	163	10999	80	278	0	0.0	0.0	14.0
Botswana	2015	8	568	66	1	0	0.0	0.0	1.0
Burkina Faso	2000	103	6796	50	1978	19	0.0	34.0	74.0
Burkina Faso	2001	165	10888	50	3123	19	0.0	34.0	74.0
Burkina Faso	2012	7	2077	314	17	2	0.0	0.4	8.0
Burundi	2007	15	1436	100	0	0	0.0	0.0	0.0
Cameroon	2003	232	13390	50	7842	33	0.0	64.1	97.4
Cameroon	2009	244	12213	50	365	1	0.0	2.0	20.9
Cameroon	2013	35	1801	47	17	0	0.0	0.0	11.1
Cape Verde	2005	16	1650	102	0	0	0.0	0.0	0.0
Central African Republic	2008	19	1310	82	144	6	0.0	8.9	49.4
Central African Republic	2009	1	142	142	101	101	71.1	71.1	71.1
Chad	2015	69	7158	100	0	0	0.0	0.0	0.0
Congo	2015	5	489	94	0	0	0.0	0.0	0.0
Congo (Brazzaville)	2008	29	2610	100	100	0	0.0	0.0	64.7
Congo (Brazzaville)	2011	9	972	110	9	1	0.0	0.9	3.6
Congo (Brazzaville)	2012	1	774	774	134	134	17.3	17.3	17.3
Congo (Brazzaville)	2013	3	256	88	0	0	0.0	0.0	0.0
Congo (Brazzaville)	2014	1	697	697	44	44	6.3	6.3	6.3
Congo (Brazzaville)	2015	149	9146	58	16	0	0.0	0.0	7.8
Congo (Kinshasa)	2010	177	17467	100	240	0	0.0	0.0	53.4
Congo (Kinshasa)	2012	91	8659	100	287	1	0.0	1.0	26.9
Congo (Kinshasa)	2013	281	26538	100	1012	0	0.0	0.0	48.0
Congo (Kinshasa)	2014	79	7944	100	320	1	0.0	1.0	64.0
Congo (Kinshasa)	2015	32	3400	100	245	0	0.0	0.0	66.0
Congo, DRC	2014	1	100	100	17	17	17.0	17.0	17.0
Cote d'Ivoire	2000	45	3248	75	226	1	0.0	1.4	70.0
Cote d'Ivoire	2001	150	10247	74	876	3	0.0	3.9	45.5
Cote d'Ivoire	2012	16	1129	59	272	18	1.8	34.3	70.0
Cote d'Ivoire	2014	8	687	93	66	6	2.3	7.4	28.0
DRC	2010	2	200	100	5	2	1.0	2.5	4.0
Equatorial Guinea	2003	42	2244	50	128	2	0.0	4.5	42.9
Equatorial Guinea	2008	13	606	50	45	2	0.0	5.3	42.9
Eritrea	2014	26	2655	100	0	0	0.0	0.0	0.0
Eritrea	2015	44	4312	100	7	0	0.0	0.0	27.3
Ethiopia	1999	1	50	50	3	3	6.0	6.0	6.0
Ethiopia	2008	35	3694	100	435	5	0.6	5.0	55.0
Ethiopia	2013	1236	122248	100	140	0	0.0	0.0	8.0
Gabon	2008	22	1453	72	66	2	0.0	3.0	29.0
Gabon	2014	208	11397	53	54	0	0.0	0.0	13.0
Ghana	1998	12	600	50	41	3	0.0	6.0	14.0
Ghana	1999	6	450	75	35	6	6.0	7.0	12.0
Ghana	2000	152	7700	50	582	3	0.0	6.0	40.0
Ghana	2001	221	17193	71	1985	4	0.0	5.1	88.0
Ghana	2002	8	861	66	75	4	0.0	5.7	22.0
Ghana	2004	4	501	105	48	14	3.0	10.9	17.1

Table SG 2: Summary of serological data used in mapping. *(continued)*

Country	Year	# survey sites	Total	Median	Total	Median	Minimum	Median	Maximum
Guinea	2005	86	8340	100	216	0	0.0	0.0	23.3
Guinea-Bissau	2004	35	3349	99	157	1	0.0	1.0	23.6
Kenya	1998	1	1013	1013	167	167	16.5	16.5	16.5
Kenya	2002	8	1447	188	501	66	27.5	33.9	42.9
Kenya	2004	1	304	304	119	119	39.1	39.1	39.1
Liberia	2006	7	651	96	51	2	0.0	2.1	34.4
Liberia	2012	23	1343	52	106	2	0.0	3.2	46.2
Madagascar	2004	204	18561	100	1753	3	0.0	3.0	58.0
Madagascar	2005	183	16835	100	1427	2	0.0	2.2	58.0
Madagascar	2015	19	1895	100	0	0	0.0	0.0	0.0
Malawi	2000	23	2149	78	1158	29	15.3	48.4	74.4
Malawi	2002	8	716	84	344	45	15.3	48.6	68.1
Malawi	2003	63	5040	78	491	6	0.0	7.4	35.8
Mali	2002	213	13779	50	4601	18	0.0	34.0	87.5
Mali	2004	1	100	100	0	0	0.0	0.0	0.0
Mauritania	2015	143	14612	101	39	0	0.0	0.0	3.5
Mozambique	2004	133	10781	100	1508	6	0.0	6.0	82.0
Mozambique	2006	221	17948	100	2633	10	0.0	11.0	82.0
Niger	2002	63	4637	75	565	10	0.0	17.0	66.0
Niger	2003	3	500	100	44	2	0.0	2.0	14.0
Niger	2007	3	1300	400	45	22	0.0	4.4	5.8
Niger	2009	2	1075	538	3	2	0.2	0.3	0.4
Niger	2012	4	1469	365	5	2	0.0	0.4	0.6
Nigeria	2000	28	2010	50	476	16	5.0	22.0	62.0
Nigeria	2002	3	259	100	10	1	0.0	1.0	15.3
Nigeria	2003	203	15273	80	737	1	0.0	2.1	26.8
Nigeria	2004	25	2230	100	164	2	0.0	2.0	66.0
Nigeria	2005	19	1648	100	121	1	0.0	1.0	58.0
Nigeria	2006	2	111	56	48	24	34.0	42.4	50.8
Nigeria	2007	21	1935	100	164	3	0.0	3.0	36.0
Nigeria	2008	419	30372	72	3500	4	0.0	7.5	88.0
Nigeria	2009	43	2995	50	196	1	0.0	2.0	47.2
Nigeria	2010	164	12605	50	696	2	0.0	2.0	58.0
Nigeria	2011	85	6305	85	132	1	0.0	2.0	14.0
Nigeria	2012	6	600	100	141	24	17.0	23.5	30.0
Nigeria	2013	55	4144	97	65	1	0.0	2.0	18.0
Rwanda	2008	25	1494	50	2	0	0.0	0.0	2.0
Sao Tome & Principe	2015	25	2132	83	31	1	0.0	1.1	7.0
Senegal	2003	50	5275	100	335	2	0.0	1.3	78.1
Senegal	2010	49	4901	100	78	0	0.0	0.0	10.0
Sierra Leone	2005	76	4507	50	906	10	0.0	20.0	68.0
South Sudan	2009	43	5254	109	4	0	0.0	0.0	1.0
South Sudan	2016	42	3586	100	31	0	0.0	0.0	7.1
Tanzania (Mainland)	1998	86	4266	50	1598	20	0.0	40.0	72.0
Tanzania (Mainland)	2000	20	950	50	504	28	0.0	55.0	72.0
Tanzania (Mainland)	2001	29	2412	100	1112	29	20.3	40.3	69.0
Tanzania (Mainland)	2002	1	907	907	395	395	43.6	43.6	43.6
Tanzania (Mainland)	2004	163	8402	51	1193	5	0.0	10.5	46.7
Tanzania (Mainland)	2005	4	578	144	106	22	0.0	11.7	62.9
Tanzania (Zanzibar)	2004	1	50	50	4	4	8.0	8.0	8.0
The Gambia	2001	1	100	100	0	0	0.0	0.0	0.0
The Gambia	2002	41	4099	100	21	0	0.0	0.0	3.0
Togo	2000	58	2900	50	58	0	0.0	0.0	36.0
Togo	2001	85	6954	84	87	0	0.0	0.0	36.0
Togo	2002	12	974	81	56	0	0.0	0.0	31.3
Uganda	1998	4	2975	893	694	212	0.0	23.7	30.1
Uganda	2002	143	32840	225	1171	0	0.0	0.0	30.7
Uganda	2003	37	3432	100	157	0	0.0	0.0	35.0
Uganda	2009	7	1606	253	26	3	0.0	1.2	3.1
Uganda	2010	25	1811	72	0	0	0.0	0.0	0.0
URT	2000	1	100	100	1	1	1.0	1.0	1.0
URT	2001	3	300	100	65	5	2.0	3.3	59.0
Zambia	2003	44	3965	91	389	0	0.0	0.0	53.9

Table SG 2: Summary of serological data used in mapping. *(continued)*

Country	Year	# survey sites	Total	Median	Total	Median	Minimum	Median	Maximum
Zambia	2004	35	3262	98	414	6	0.0	6.0	53.9
Zambia	2005	29	2759	100	331	11	0.0	12.0	30.0
Zambia	2009	36	2855	98	136	2	0.0	2.7	20.8
Zambia	2010	77	7105	100	366	4	0.0	4.0	20.8
Zambia	2011	2	647	324	48	24	1.0	4.8	8.6
Zimbabwe	2014	111	9858	100	77	0	0.0	0.0	10.0

2 Figures

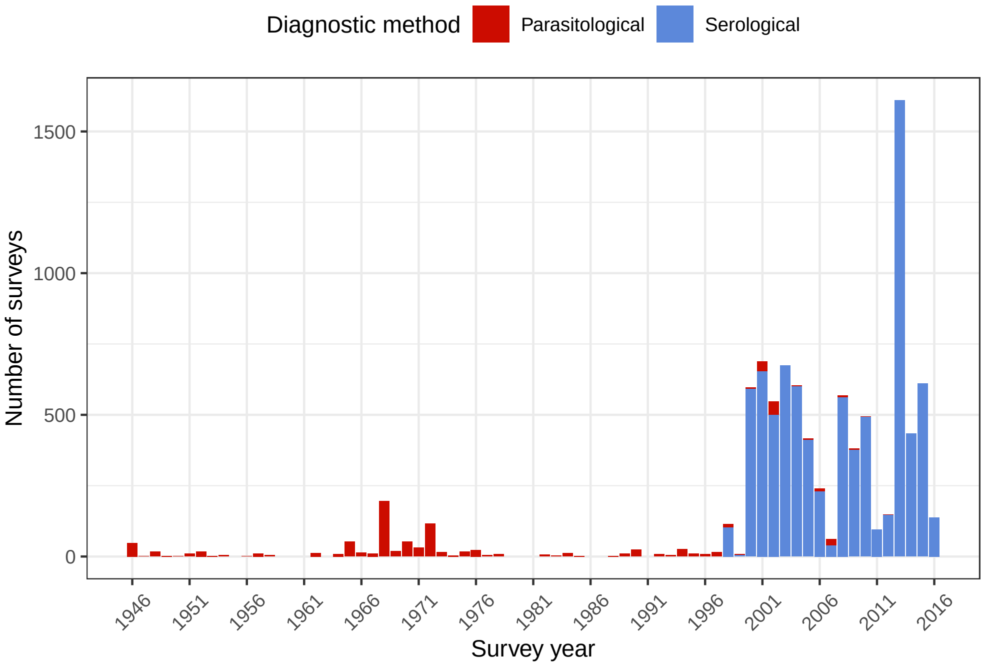

Figure SG 1: Time distribution of surveys by diagnostic method.

Table SG 3: Monte Carlo maximum likelihood estimates and corresponding 95% confidence intervals for the model fitted to lf prevalence data.

Parameter	Estimate	95% CI
β_0	0.016	(0.011, 0.022)
β_1	1.155	(0.991, 1.347)
β_2	1.011	(0.89, 1.148)
β_3	1.390	(1.22, 1.583)
β_4	1.112	(0.932, 1.326)
β_5	1.152	(0.953, 1.392)
β_6	1.262	(0.97, 1.642)
β_7	0.995	(0.881, 1.124)
β_8	0.955	(0.857, 1.064)
β_9	1.071	(0.961, 1.194)
β_{10}	1.003	(0.971, 1.036)
β_{11}	1.008	(0.971, 1.046)
β_{12}	1.076	(0.985, 1.175)
β_{13}	0.909	(0.812, 1.017)
β_{14}	1.082	(0.975, 1.2)
β_{15}	0.969	(0.892, 1.052)
β_{16}	1.016	(0.925, 1.115)
σ^2	5.254	(4.694, 5.88)
Practical range	394.163	(344.082, 451.533)
τ^2	0.572	(0.448, 0.731)
α	0.505	(0.498, 0.513)

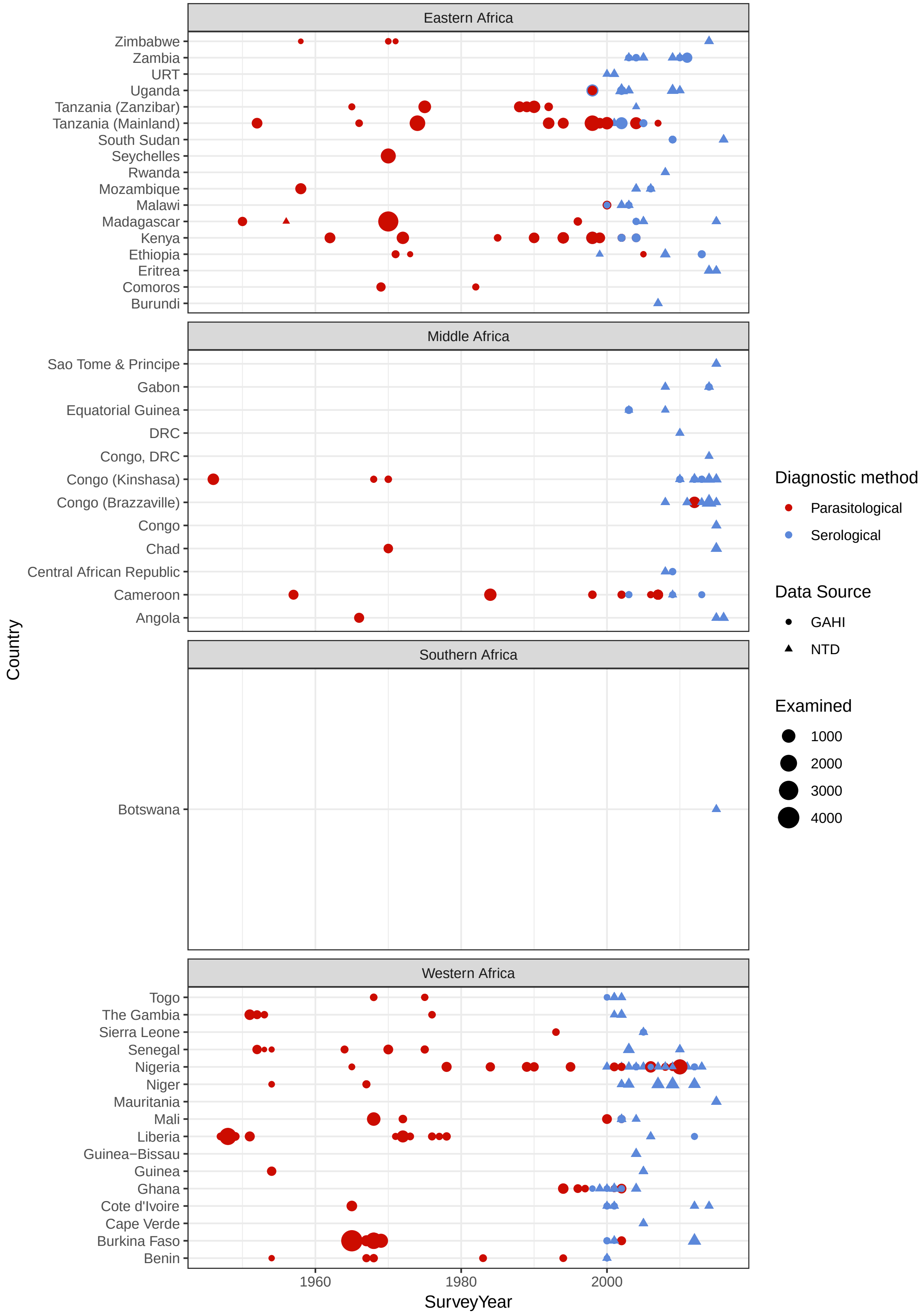

Figure SG 2: Data availability by survey size, type and country

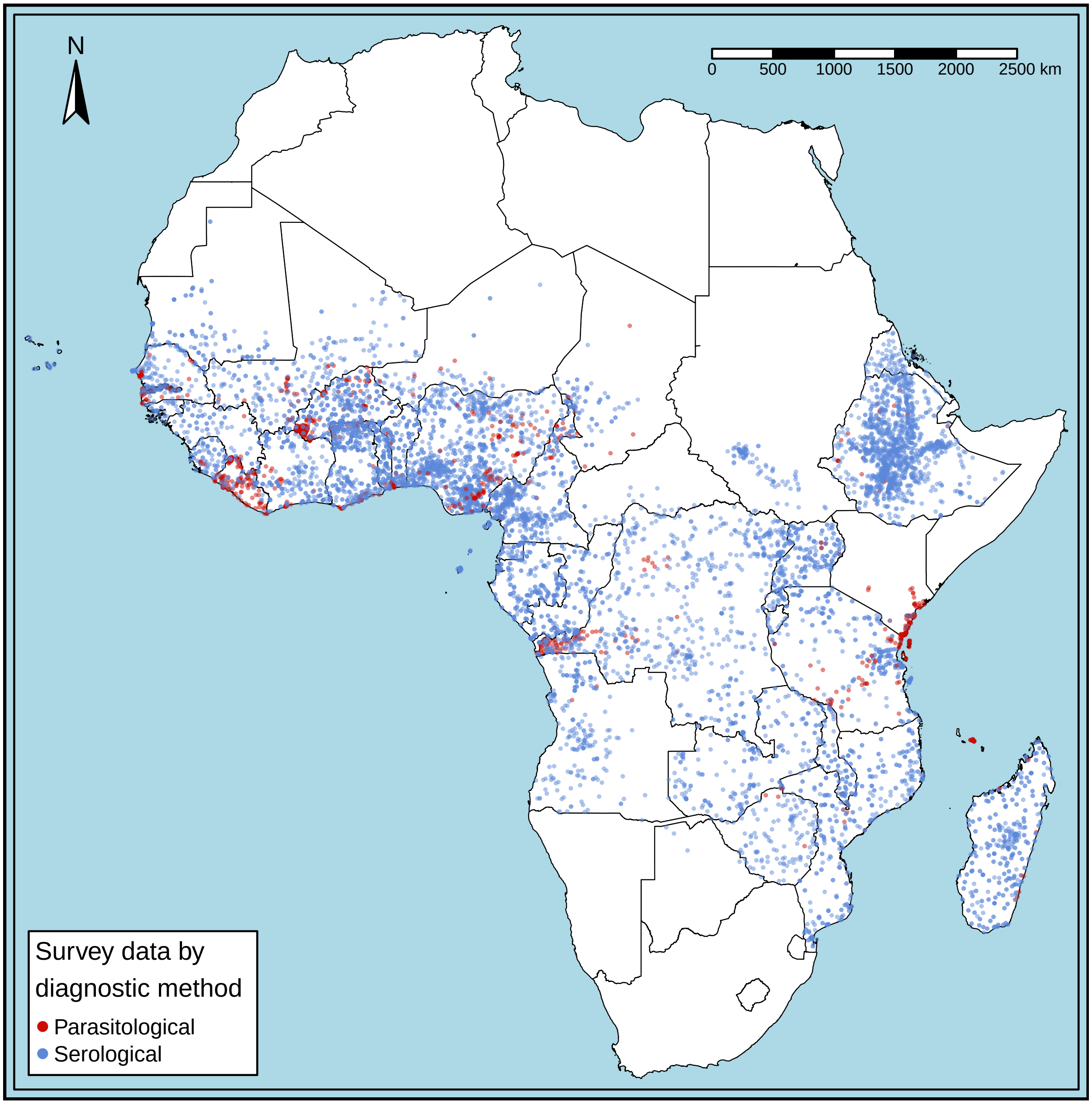

Figure SG 3: Spatial distribution of surveys by diagnostic method.

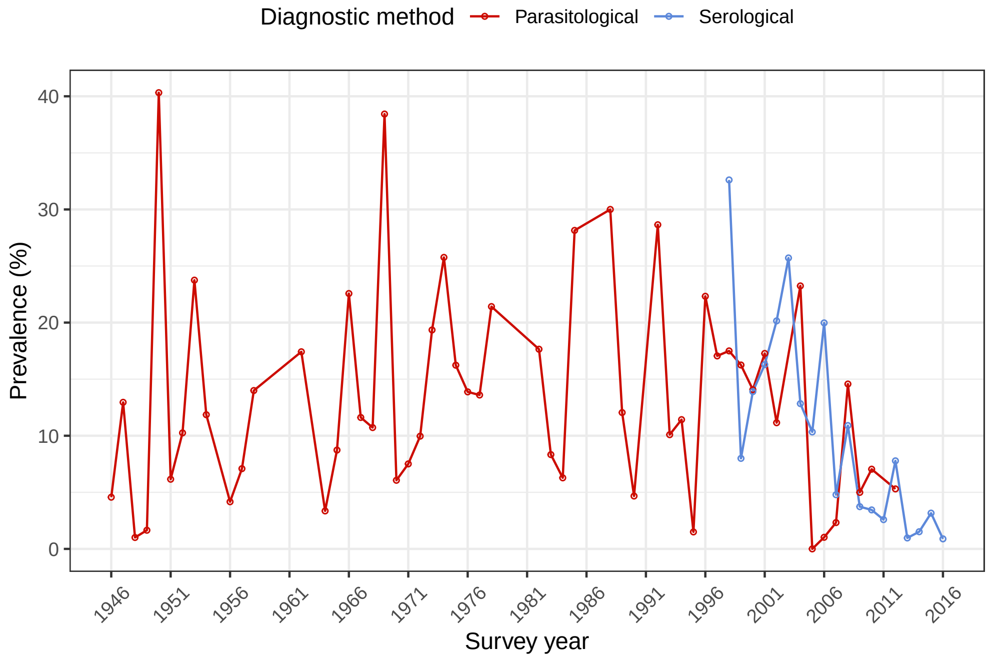

Figure SG 4: Time distribution of prevalence averaged by year.

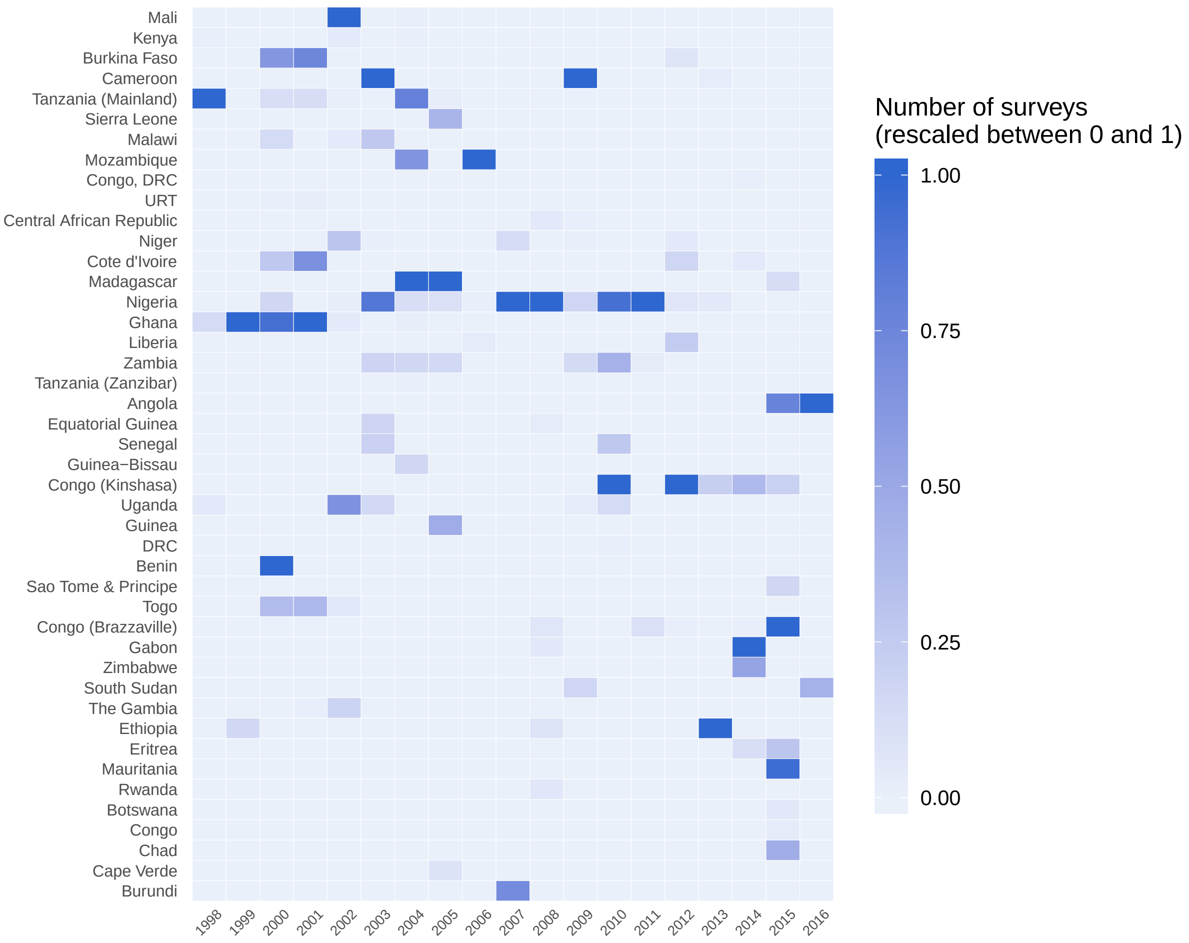

Figure SG 5: Spatial and temporal distribution of ICT mapping surveys. The countries are ordered from the most endemic to the less endemic.

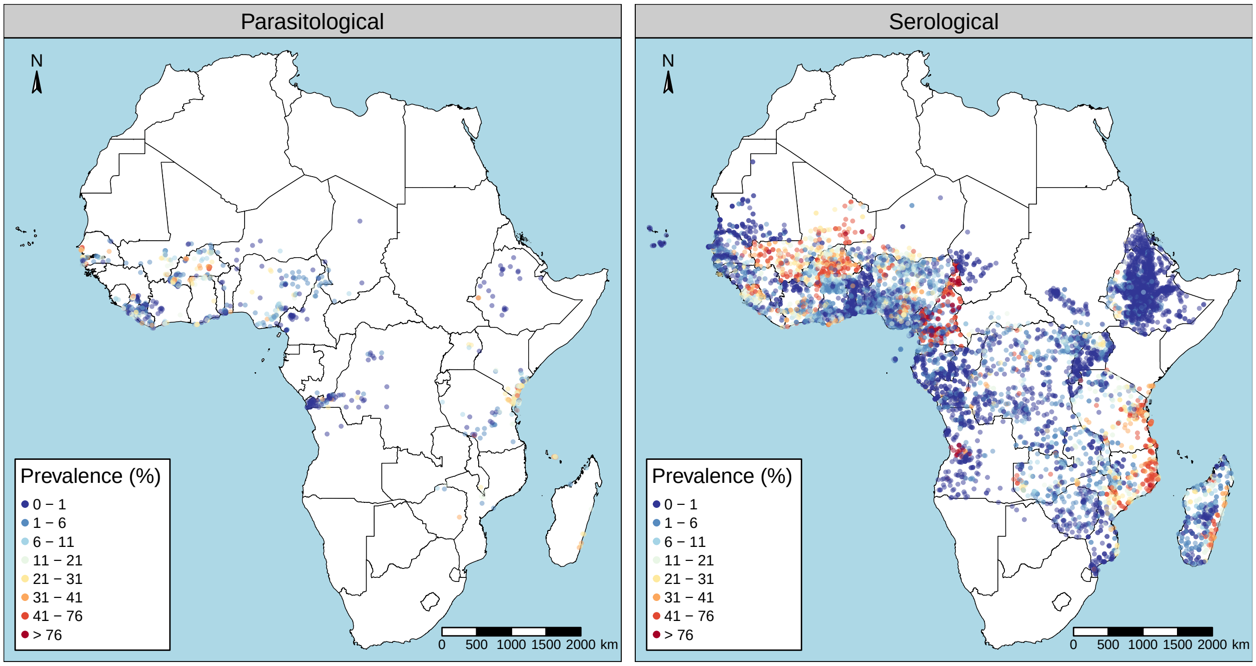

Figure SG 6: The spatial distribution of data on the prevalence of microfilaraemia (parasitological) and antigenaemia (serological), based on immuno-chromatographic card test (ICT).

```
## variog: co-locatted data found, adding one bin at the origin
```

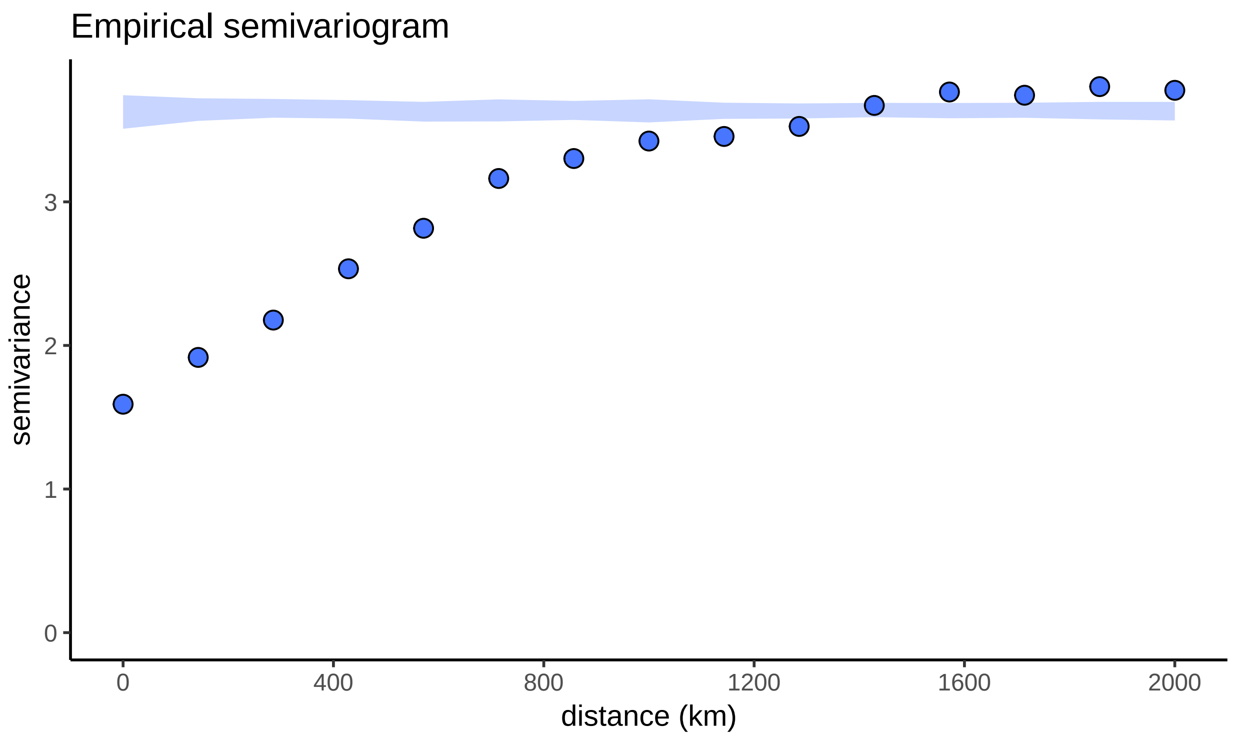

Figure SG 7: Empirical semivariogram for microfilaraemia (parasitological) and antigenaemia (serological) logit prevalence.

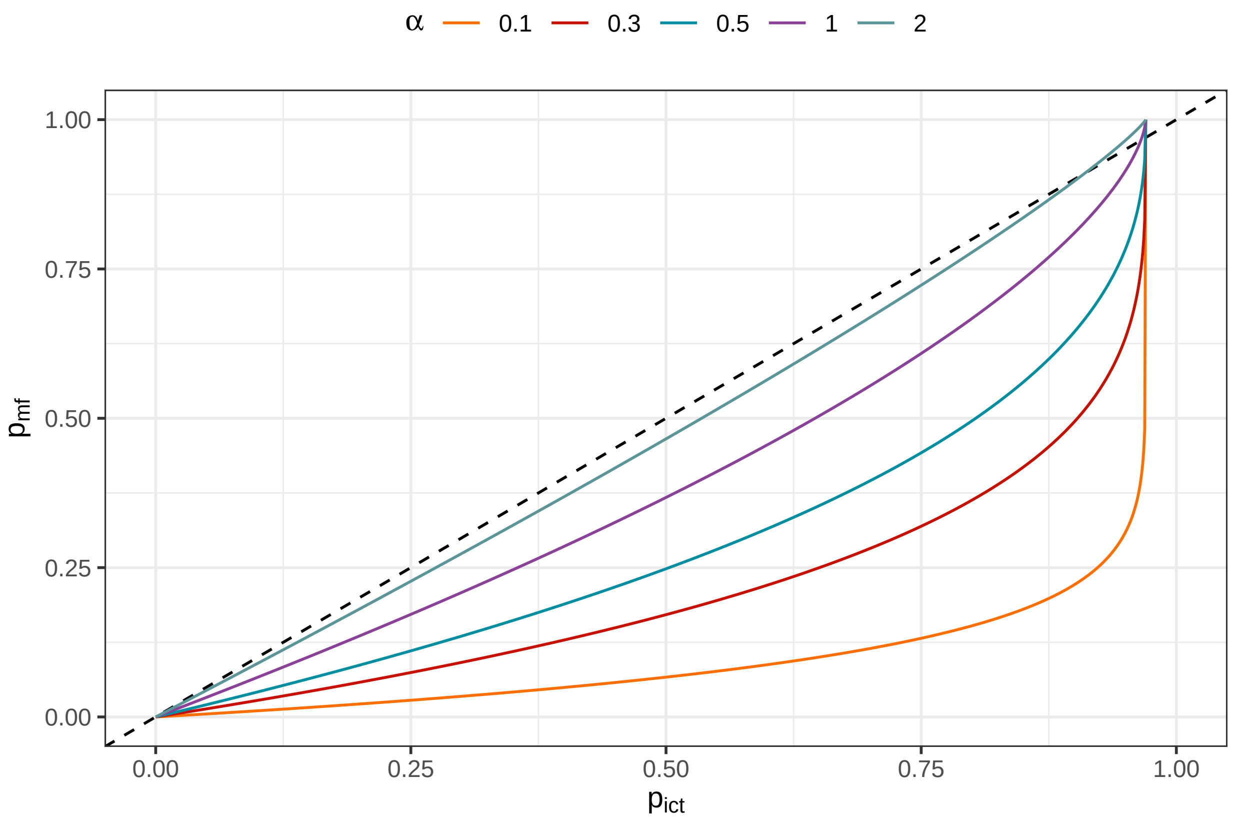

Figure SG 8: Mechanistic relationship betwen ict and mf prevalence for different levels of α. The black dashed line is the bisector of the first quadrant.

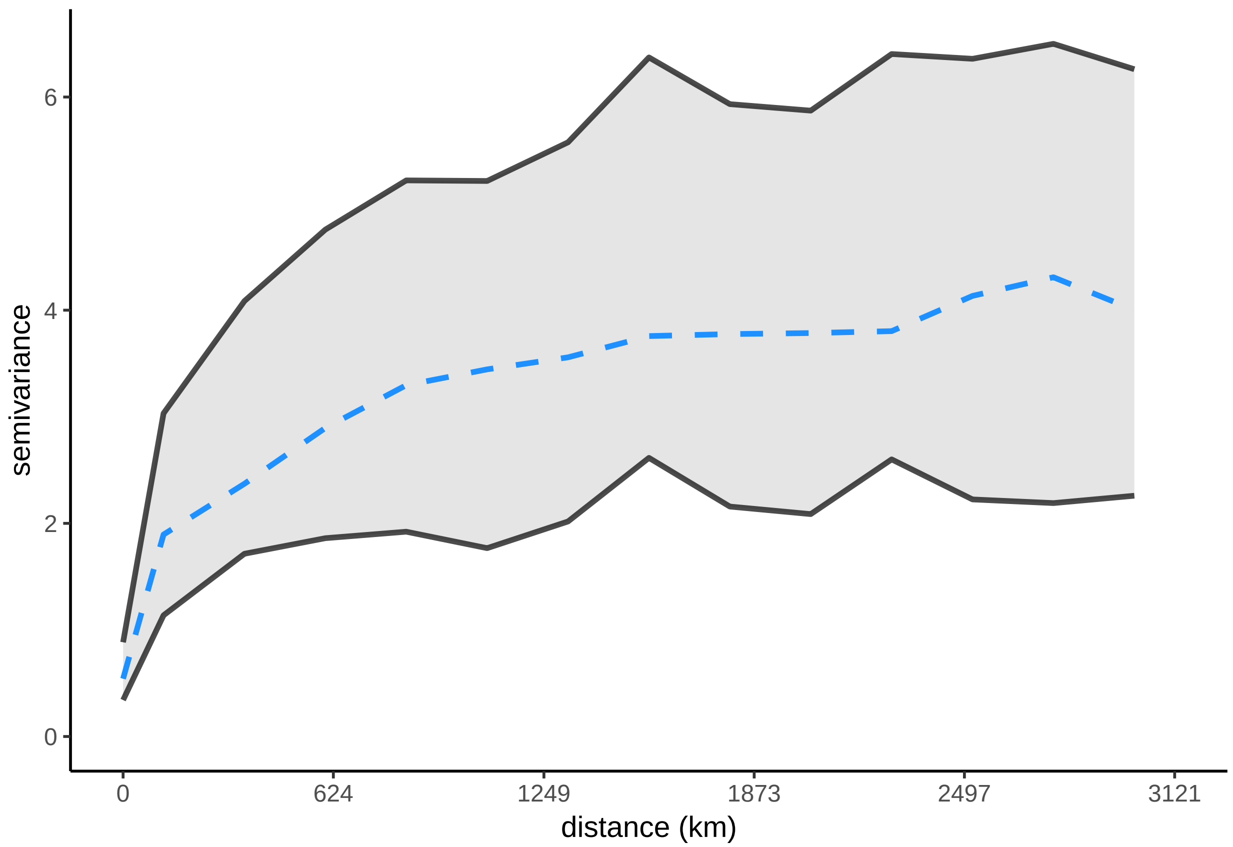

Figure SG 9: Empirical variogram (dashed line) and 95% confidence bands (solid lines) from 100 data set simulated from the fitted model.

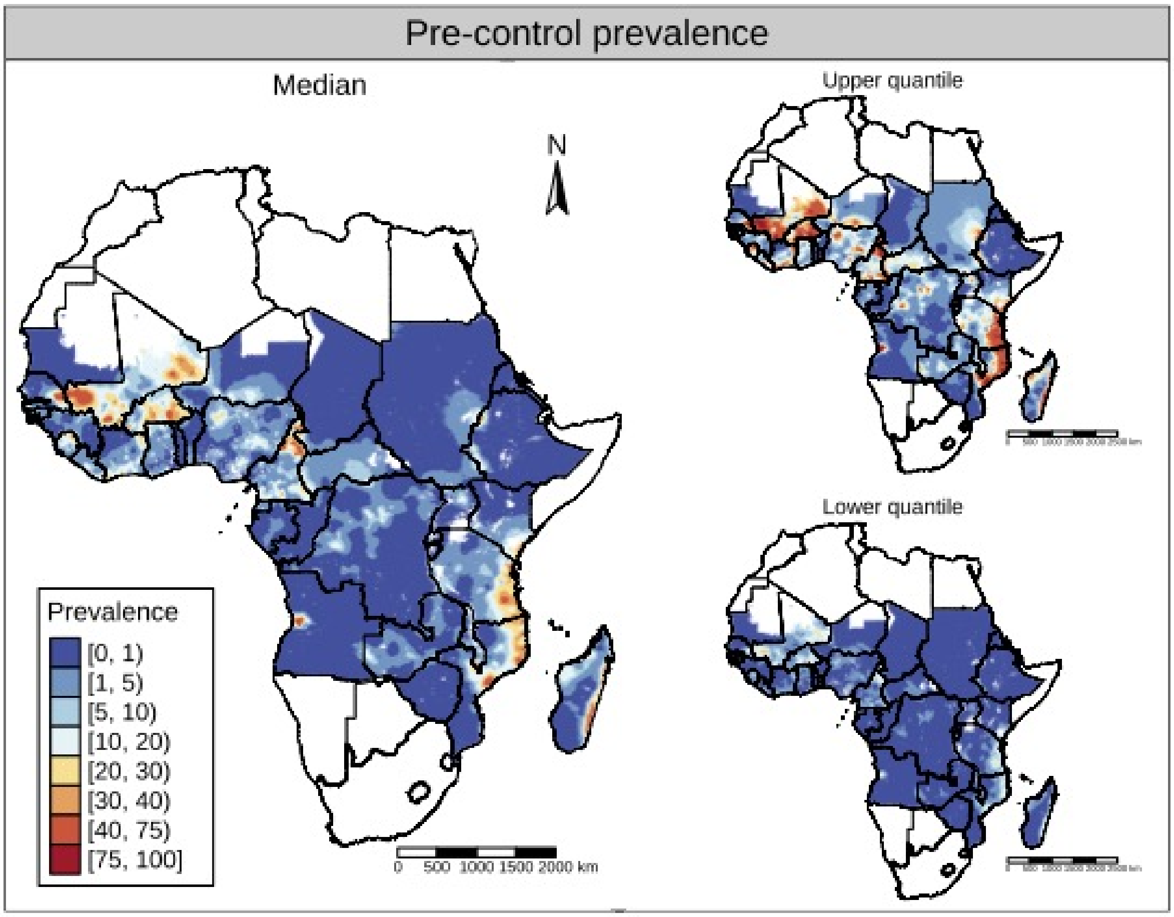

Figure SG 10: Median pre-control geostatistical map, together with the lower (2.5%) and upper (97.5%) quantiles.
